# Genetics and genomics of Parkinson’s disease

**DOI:** 10.1186/gm566

**Published:** 2014-06-30

**Authors:** Michelle K Lin, Matthew J Farrer

**Affiliations:** 1 Djavad Mowafaghian Centre for Brain Health, Centre for Applied Neurogenetics, Department of Medical Genetics, University of British Columbia, Vancouver, BC V6T 1Z3, Canada

## Abstract

Parkinson’s disease (PD) is a progressively debilitating neurodegenerative syndrome. Although best described as a movement disorder, the condition has prominent autonomic, cognitive, psychiatric, sensory and sleep components. Striatal dopaminergic innervation and nigral neurons are progressively lost, with associated Lewy pathology readily apparent on autopsy. Nevertheless, knowledge of the molecular events leading to this pathophysiology is limited. Current therapies offer symptomatic benefit but they fail to slow progression and patients continue to deteriorate. Recent discoveries in sporadic, Mendelian and more complex forms of parkinsonism provide novel insight into disease etiology; 28 genes, including those encoding alpha-synuclein (*SNCA*), leucine-rich repeat kinase 2 (*LRRK2*) and microtubule-associated protein tau (*MAPT*), have been linked and/or associated with PD. A consensus regarding the affected biological pathways and molecular processes has also started to emerge. In early-onset and more a typical PD, deficits in mitophagy pathways and lysosomal function appear to be prominent. By contrast, in more typical late-onset PD, chronic, albeit subtle, dysfunction in synaptic transmission, early endosomal trafficking and receptor recycling, as well as chaperone-mediated autophagy, provide a unifying synthesis of the molecular pathways involved. Disease-modification (neuroprotection) is no longer such an elusive goal given the unparalleled opportunity for diagnosis, translational neuroscience and therapeutic development provided by genetic discovery.

## Clinical features, main treatments and challenges

Parkinson’s disease (PD) is a progressively debilitating neurodegenerative disease that becomes increasingly challenging to manage. Until recently, the underlying molecular cause(s) have remained elusive, as have hopes of disease-modification (neuroprotection). The need for advances in understanding and treating the disease is great as PD is the second most common neurodegenerative disorder after Alzheimer’s disease. The estimated worldwide prevalence is 1% in the population >60 years of age, increasing to 4% at 80 years of age [1]. The median age of onset is about 70 years but about 4% of patients manifest early-onset disease (before they reach 50 years of age).

In PD, the clinical heterogeneity, disease course and response to medication vary widely [2]. Motor symptoms are associated with profound neuronal loss in the *substantia nigra pars compacta* (SN), depleting the striatum of dopaminergic inhibition. Hence, restorative dopaminergic therapies are the main treatment. Monoamine oxidase inhibitors are initially used to prevent endogenous dopamine catabolism; alternatively, L-DOPA, the metabolic precursor of dopamine, and/or dopamine agonists are used. In selected patients, deep brain stimulation (DBS) of striatal output pathways has also proven effective [3]. There are, however, several non-motor symptoms, of which many are non-dopaminergic and without remedy [4]. For example, 30% of patients develop mild-cognitive impairment within 5 years of motor symptoms and many develop dementia [5]. PD is clinically and/or pathologically distinct from other forms of parkinsonism (Box 1). A definitive diagnosis of PD requires the presence of Lewy bodies and Lewy neurites (proteinaceous intracellular inclusions) in the brain stem (midbrain), although these lesions are often more widespread [6,7]. Here, we review recent genetic and genomic findings in studies of PD, we provide some integration and synthesis of the molecular pathways involved, and we discuss the translational implications.

## Early linkage and candidate gene studies

Classical linkage analysis has proven a powerful approach for the identification of specific disease-associated genes and mutations in families with multi-incident parkinsonism [8]. Genome-wide association and twin studies further demonstrate that even idiopathic sporadic PD has a significant genetic component [9,10]. Mutant gene discovery by linkage with association provides an unequivocal burden of proof, and is the foundation required for translational neuroscience. Nevertheless, genetically defined carriers may have variable expressivity and penetrance and may never ‘phenoconvert’ to symptomatic disease. Age remains an important determinant, even in families with dominant, recessive or X-linked patterns of segregation. Not surprisingly, *in vivo* modeling in mammalian systems is challenging if the expectation is to recapitulate the human phenotype. Gene discovery efforts in PD have been expertly reviewed and we provide some historical context in Box 2. In this review, we focus on more typical, late-onset PD with Lewy body pathology, the disease type suffered by the majority of patients, and suggest how recent discoveries might unify existing ideas to suggest novel pathways and therapeutic targets.

In this context of genomic discovery, genes at three loci - alpha-synuclein (*SNCA*), leucine-rich repeat kinase 2 (*LRRK2*) and microtubule-associated protein tau (*MAPT*) - deserve special mention, although the molecular relationship between them has yet to be elucidated. Both *SNCA* and *LRRK2* assignments were originally implicated by the discovery (by linkage analysis) of pathogenic mutations that segregate within families, and these observations were extended into idiopathic PD by candidate gene studies. Combined pooled analysis by the Genetic Epidemiology of Parkinson’s disease Consortium (http://www.geopd.org), literature meta-analysis (http://www.pdgene.org) and more recent genome-wide association studies (GWAS) have provided compelling support for the involvement of these loci.

Missense and multiplication mutations (duplication and triplication) in *SNCA* lead to PD, subsequent dementia and fulminant diffuse Lewy body disease on autopsy [11]. Levels of gene expression are inversely correlated with age at symptom onset. In rodents, *SNCA* overexpression may recapitulate many of the features of PD, whereas knockout mice are viable and fertile and appear to have little sign of disease [12]. The alpha-synuclein protein promotes presynaptic SNARE complex assembly, synaptic vesicle exocytosis and reciprocal plasmalemma endocytosis [13]. Alpha-synuclein protein aggregates, however, may also behave pathologically as prion proteins [14]. Transplants of fetal tissue into the striatum of human patients have been observed to develop Lewy-body pathology [15]. Similarly, iatrogenic inoculation of alpha-synuclein oligomers into mouse brain leads to widespread Lewy-like pathology, albeit requiring endogenous alpha-synuclein for transmission [16]. Hence, down-regulation or suppression of *SNCA* may represent one mechanism to slow disease progression [17]. Similarly, therapies that enhance the clearance of Lewy aggregates, including immunotherapies targeting potentially toxic forms of alpha-synuclein, might be neuroprotective [18].

*LRRK2* parkinsonism is clinically indistinguishable from idiopathic PD. The age of onset and age-dependent cumulative incidence is similarly broad, although disease progression in carriers of *LRRK2* mutations is more homogeneous [19]. A founder haplotype has been noted for *LRRK2* p.G2019S in most populations. Penetrance appears to be ethnic-specific with a lifetime cumulative risk of parkinsonism in p.G2019S carriers being 22% for Ashkenazi Jews (living in the US), 45% for Norwegians and 80% for Arab-Berbers [20-22], which is an important consideration for genetic counseling. For Ashkenazi Jews in Israel and Arab-Berbers in Tunisia the penetrance of disease is similar. In these countries, the frequency *of LRRK2* p.G2019S carriers in healthy controls is relatively high at 1 to 2% [23,24]. While genetic drift may be sufficient explanation for the high frequency of *LRRK2* p.G2019S carriers in healthy controls, positive selection may also contribute; *LRRK2* is associated with intestinal inflammatory disorders, immune response and kidney function, which if compromised may be most deleterious in hot climates [25-27]. Overall, only a small proportion (approximately 1%) of familial and sporadic PD is likely explained by *LRRK2* p.G2019S; other variability, such as the p.G2385R mutation, which is frequent throughout Asia, most significantly contributes to population-attributable risk [28] (Table 1).

**Table 1:** Genomic loci implicated in Parkinson’s disease by genome-wide association analyses

**Gene**	**Chr**	**Associated** **SNP/locus**	**Genes within locus**^**a**^	**Odds ratio** **[95% CI]**	***P*-value**
*GBA*	1q21	N370S	*TRIM46*, *MUC1*, *MIR92B*, *THBS3*, *GBAP1*-*GBA*-*FAM189B*, *SCAMP3*, *CLK2*, *HCN3*, *PKLR*	3.37 [2.67-4.29]	1.11E-24
*SYT11/RAB25*	1q21	chr1:154105678	*MIR7851*, *UBQLN4*, *LAMTOR2*-*RAB25*-*MEX3A*, *LMNA*	1.67 [1.41-1.98]	5.70E-09
*PM20D1*	1q32	rs11240572	*NUCK1*-*RAB7L1*-*SLC41A1*, *PM20D1*	0.74 [0.69-0.80]	1.01E-14
*STK39*	2q24	rs2102808	*STK39*	1.28 [1.19-1.38]	1.54E-11
*MCCC1/LAMP3*	3q27	rs11711441	*MCCC1*-*LAMP3*-*MCF2L2*	0.84 [0.80-0.89]	8.72E-12
*BST1*	4p15	rs4698412	*FAM200B*-*BST1*	0.87 [0.83-0.91]	2.28E-10
*GAK/DGKQ*	4p16	rs1564282	*CPLX1*-*GAK*-*TMEM175*-*DGKQ*-*SLC26A1*, *IDUA*, *FGFRL1*	1.29 [1.20-1.38]	6.54E-13
*SNCA*	4q21	rs356220	*SNCA*-*MMRN1*	1.30 [1.25-1.34]	3.06E-49
*HLA-DRB5*	6p21	rs2395163	*HLA-DRB5*-*HLA-DRB1*, *HLA-DRB6*	0.75 [0.68-0.84]	2.90E-07
*GPNMB*	7p15	rs156429	*GPNMB-MALSU1-IGF2BP3*	0.89 [0.86-0.93]	2.69E-10
*LRRK2*	12q12	rs34778348	*SLC2A13*-*LRRK2*-*MUC19*, *CNTN1*	2.23 [1.89-2.63]	2.97E-21
*CCDC62/HIP1R*	12q24	rs12817488	*DENR*-*HIP1R*-*VPS37B*, *ABCB9*, *OGFOD2*,	1.17 [1.09-1.25]	2.99E-06
*MAPT/STH*	17q21	H1H2, 900kb inversion	*ARHGAP27*, *PLEKHM1*, *CRHR1*, *SPPL2C*-*MAPT*-*STH*, *KANSL1*, *LRRC37A*, *NSFP1*, *ARL17A/B*	0.78 [0.75-0.80]	3.54E-52

Chr, chromosomal band; CI, confidence interval. ^a^Genes within 100 kb of the most significantly associated SNP annotated from the UCSC genome browser (hg19). Odds ratios and *P*-values are the most significant findings from the PDGene database [150].

At post-mortem, the majority of patients with pathogenic *LRRK2* mutations have Lewy-body disease [29]. However, even within families with the same mutation, pleomorphic pathologies have been observed. These include neurofibrillary tangles and tufted astrocytes (4R-tauopathies, as inclusion of *MAPT* exon 10 leads to the production of tau protein with four microtubule-binding domains), Tar DNA-binding protein 43 and ubiquitin immune-positive aggregates, or simply nigral neuronal loss with gliosis [30-32]. The variable penetrance and alternative end-stage pathologies most likely reflect genetic and/or environmental modifiers and stochastic factors, and have yet to be defined. Nevertheless, genetically defined cohorts of patients with parkinsonism such as LRRK2 p.G2019S parkinsonism might allow the identification of biomarkers of disease progression and inform clinical trials [19].

As the most common genetic cause of PD, LRRK2 and its protein interactions are a logical place to search for novel therapeutic targets. The domains of LRRK2 include armadillo and ankyrin repeat regions, leucine-rich repeat (LRR), Ras of complex GTPase (Roc), C-terminal of Ras (COR), kinase and WD40. LRRK2 is a ROCO protein, with a Ras GTPase and a kinase in one molecule; these activities have established roles in other organisms or cell types in dynamically modifying the actin cytoskeleton [33]. Pathogenic *LRRK2* mutations are primarily found in the GTPase Roc domain (p.R1437H, p.R1441C/G/H), the kinase domain (p.G2019S, p.I2020T) and intervening C-terminal of Roc (p.Y1699C), whereas susceptibility variants may also be found in protein-protein interaction domains (WD40 p.G2385R) [34]. Competitive inhibition of LRRK2 kinase is presently considered as one therapeutic target given that the p.G2019S mutation activates kinase activity, autophosphorylation and/or phosphorylation of substrates [35]. However, data on the first identified substrate, moesin, which is a filamentous actin tether, have not been recapitulated *ex* or *in vivo*[36]. LRRK2 protein levels also diminish with aging, with knock-in of *LRRK2* pathogenic mutations into the mouse genome [26] and with kinase inhibition [37,38]. LRRK2 probably functions as a dimer or higher molecular weight scaffold, with many protein-protein interactions. The activities of the Roc, COR and kinase domains are interconnected [39], and the many physiologic functions of the protein complex have yet to be fully elucidated.

An association between PD and *MAPT* and the surrounding 17q21 locus results from an ancient paracentric inversion and was robustly implicated in clinical PD and in autopsy-confirmed series of PD in Caucasian populations [40-42]. Of note, similar variability in the *MAPT* locus has been unequivocally implicated in progressive supranuclear palsy, but not in Alzheimer’s disease, although tau is a major component of the neurofibrillary tangle pathology in both conditions [43]. Splice and missense mutations were first described in frontotemporal dementia [44] and the inversion region was subsequently implicated in 17q21.31 microdeletion syndromes [45], but neither pathologic mutations nor tau pathology are found in PD. Thus, genetic variability in neighboring genes, within or flanking the *MAPT* inversion, may contribute. Some examples of additional genes and pathogenic mutations discovered through family-based linkage analysis of parkinsonism are summarized in Table 2.

**Table 2:** Mendelian mutations in familial parkinsonism

**Gene**	**Mutation(s)**	**OMIM**	**Reference(s)**
**Dominantly inherited, late onset parkinsonism with Lewy pathology**			
*SNCA*	Locus multiplication and missense mutations: A30P, E46K, H50Q, G51N, A53T	*168601*, *605543*	[56,58]
*LRRK2*	R1437H, R1441H, R1441G, R1441C, Y1699C, G2019S, I2020T	*607060*	[20,31,32]
*VPS35*	D620N	*614203*	[63,64]
*EIF4G1*	R1205H	*614251*	[149]
*DNAJC13*	N855S	*614334*	[93]
**Recessively inherited, early-onset or X-linked atypical parkinsonism**			
*PARK2 (Parkin)*	Numerous exon deletions, duplications and missense mutations	*600116*	[109]
*PINK1*	Rare locus and exon deletions. Numerous missense mutations, including E129X, Q129fsX157, P196L, G309N W437X, G440E, Q456X	*605909*	[147,151]
*DJ-1*	Deletions and missense: dup168-185, A39S, E64D, D149A, Q163L, L166P, M261I.	*606324*	[123]
*DNAJC6*	Splice site c.801 -2 A > G and truncating mutation Q734X	*615528*	[70]
*ATP13A2*	Missense: L552fsX788, M810R, G877R, G1019fsX1021. Small insertions and deletions: 1103insGA, del2742TT	*606693*	[102]
*FBXO7*	T22M, R378G, R498X	*260300*	[68]
*PLA2G6*	D331Y, R635Q,R741Q, R747W	*612953*	[152]
*ATP6AP2*	Splice site mutations		[71]
*SYNJ1*	Homozygous missense: R258Q	*615530*	[72]

OMIM, Online Mendelian Inheritance in Man, a database that catalogs all the known diseases with a genetic component.

## Genome-wide association studies

While there are several genetic models for disease susceptibility, the ‘rare variant common disease’ model largely explains Mendelian heritability (one allele of major effect segregating with familial disease) whereas the ‘common variant common disease’ model (or multiple rare variants on common ancestral haplotypes) forms the theoretical basis of GWAS [46]. Built upon large, multi-institutional consortia, GWAS have dominated the search for novel genes in human traits in recent years. Collectively, more than 2,600 genomic regions (loci) of modest effect size (odds ratio <1.5) have been associated with >350 complex traits and have implicated underlying genes that play a role in disease causality or susceptibility [47]. Genome-wide genotyping, with approximately 0.5 to 1 million markers, is used to assess frequency differences in case-control designs, and is able to capture common genetic contributions to disease in linkage disequilibrium.

The first GWAS for PD were small and underpowered, showing little overlap in results [48,49]. Subsequently, there was an appreciation that larger numbers of markers and subjects are required for meaningful discovery and replication. Past North American and European efforts included analysis of familial parkinsonism [50] and of unrelated case-control series [51-54]. A web-based ‘direct- to-consumer’ effort, which is based on self report rather than clinical exam, is by far the largest study to date [9]. However, genomic imputation of single nucleotide polymorphisms (SNPs) in linkage disequilibrium allows datasets to be combined. In 2011, the International Parkinson Disease Genomics Consortium conducted a meta-analysis from five GWAS datasets [55]. Over 7 million SNPs based on approximately 1 million genotypes per individual were imputed *in silico*: the data were collected from 5,333 cases and 12,019 controls in the discovery phase, followed by 7,053 cases and 9,007 controls in the replication phase. Six loci previously associated with idiopathic PD were replicated and five new loci were identified (Table 1). Data from the latest mega-meta-GWAS, including over 13,000 patients with PD and 80,000 control subjects, are eagerly awaited. Nevertheless, in the same Caucasian populations, the genetic variance explained is unlikely to increase in proportion to the sample size and investment. While additional loci may be nominated, these assignments are likely to be of smaller effect and will require independent validation.

Overall, the most significant GWAS associations are at chromosomal bands 4q22 and 17q21 and support *SNCA* and *MAPT* assignments, which were first identified in linkage and candidate gene studies [41,56-58]. Nevertheless, GWAS illustrates that the etiology of PD is genetically heterogeneous and novel loci may yet be identified, especially within under-represented non-Caucasian populations; for example, *PARK16, BST1* and *SYT11* were identified in a Japanese study [52]. Although a risk allele identified in one population should be a risk allele for all (the genotypic-attributable risk), their frequencies may be ethnically specific and lead to widely divergent population-attributable risks. For example, Caucasians have two major haplotypes (H1 and H2) for the *MAPT* locus; only H1 is present in Asian populations whereas the frequency of H2 is about 20% in Caucasians. The *MAPT* locus is significantly associated with PD in Caucasian studies but does not appear in a Japanese GWAS [51,52]. Conversely, the *PM20D1* locus was most clearly associated with PD in Japanese GWAS (5 to 8% differences between cases and controls) [52], whereas *PM20D1* allele frequencies in Caucasian studies were similar in cases and controls (1% difference) [51].

It is important to note that genomic loci are not disease genes *per se*. Within each genomic locus, there may be numerous genes of which one or all may be candidates contributing to disease risk. An illustration is the *GAK-DGKQ* locus that is significantly associated with PD [9,50,55]. *GAK* and *DGKQ* are in complete linkage disequilibrium and both proteins have important roles in clathrin-mediated vesicular trafficking [59,60]. Furthermore, no coding mutations have been identified in any GWAS locus or gene except for those previously identified through linkage studies in families (that is, *LRRK2* and *SNCA*). Rather, these associations are ascribed to subtle differences in wild-type gene expression, for which RNA silencing and overexpression models may be informative. Common variants of modest effect, on common haplotypes, may lead to modest transcriptional or functional changes. Directly genotyping a ‘causal variant’ will provide the greatest odds ratio for disease association and provides the rationale for locus-specific sequencing and further association testing. For some disease-associated loci, multiple rare variants of major effect may be responsible, in aggregate, although they will have occurred on the most frequent haplotypes.

## Next-generation sequencing for PD

Most of the genetic variants in the human genome are a consequence of mutation with recent population expansion and are present at very low frequencies (<0.5%) [61]. Collectively, rare variants are more common than frequent variants in any given population and each individual has many unique *de novo* point mutations. If these cluster within specific genomic loci in patients with a disease such as PD, they highlight genes or mutational hotspots likely to confer disease susceptibility. To Sanger sequence gene-by-gene in search of a causative variant for PD is a time-consuming and cost-limited effort. Nevertheless, the detection of rare and unique variants via direct sequencing has become more affordable with the advancement of next-generation methods.

Within families, whole-exome sequencing (WES) has proven to be effective in uncovering rare causal mutations of major effect in small sample sizes, and is considerably less expensive than whole-genome sequencing. The first proof-of-concept work discovered pathogenic variants in Freeman-Sheldon syndrome in just four unrelated affected individuals [62]. The first WES study of parkinsonism revealed the p.D620N mutation in *VPS35* (the vacuolar sorting protein 35 gene) by sequencing affected cousin-pairs in autosomal dominant kindreds with late-onset disease [63,64]. These findings have been confirmed worldwide, suggesting that *VPS35* contributes to approximately 1% of familial parkinsonism and 0.2% of sporadic PD [65-67]. WES has become the fastest method for the identification of novel genes in parkinsonism, contributing to the discoveries of *FBXO7*, *WRD45*, *DNAJC6*, *DNAJC13*, *ATP6AP2* and *SYNJ1* in recent years [68-72].

Variants in non-coding and highly conserved genomic regions may also contribute to risk, and might explain the ‘missing heritability’ underlying complex trait disorders. Whole-genome sequencing (WGS) now enables the sequencing of untranslated regions (UTRs), including gene promoters, enhancers, introns, and 5′ and 3′ UTRs. These contribute to the regulation of gene expression directly through transcription-factor binding, via microRNA and noncoding RNA mechanisms and through alternative exon splicing, and can influence the phenotypic variance of some traits. Two examples relevant to parkinsonism include 5′ UTR expansions in *FMR1* in Fragile X-associated tremor-ataxia syndrome [73] and non-coding mutations in *ATP6AP2* that contribute to aberrant alternative splicing [71].

Next-generation DNA-sequencing panels can be used for novel mutation discovery in a locus-centric approach. In genome-wide family-based studies, when seeking to identify novel genes, it is prudent to examine and exclude those genes already implicated in parkinsonism (including developmental and aging syndromes as described earlier). Unique probes or amplicons can cover exonic regions or span entire loci, and target DNA can be barcoded and sequenced in parallel for multiple individuals. Such panels have many benefits: first, they provide affordable and rapid genetic diagnoses to better inform patient treatment, without problems arising from incidental findings [74]; second, they allow the discovery of as-yet-unknown mutations in genes known to be related to PD; and third, in aggregate, the results nominate genetic variants that may further explain the heterogeneity of clinical and pathologic presentations. Similar bioinformatic filters might be applied to WES or WGS *in silico*. In a gene-centric approach, with a limited number of genes studied per assay relative to WES or WGS, the interpretation is simplified. Nevertheless, custom capture or amplification methods can produce artifacts as oligomers may not be perfectly complementary to the human genome being interrogated, and this may introduce some allelic bias.

Another pitfall of next-generation sequencing (NGS) methods is the inability to sequence repetitive regions and structural variations. For example, *SNCA* multiplications, the *GBA* loci versus its pseudogene, or indeed any repetitive regions may confound sequence analysis; most sequence read lengths are relatively short (approximately 100 to 200 bp) and may be misaligned as a consequence. Almost half of the human genome consists of repeats that play an active role in genome evolution, although large structural rearrangements may result in disease [75]. Inadequate sequence read-depth may also lead to annotation errors. Most publically shared WGS has been performed at relatively low read depths; for example, the 1000 Genomes effort achieved 2x to 6x coverage (http://www.1000genomes.org). For WES, >100x coverage is considered necessary for diagnostic testing [76]. Nevertheless, specific NGS results should be validated by Sanger sequencing to prevent false positives. Higher-coverage sequencing, longer read lengths and innovative bioinformatic approaches continue to offer significant improvements.

In studies involving WES or WGS of familial PD, rare variants can be prioritized for validation and replication in additional samples by looking at the intersection of those shared by affected family members and not shared by elderly unaffected relatives. Typically, in approximately 58 Mb of exome sequencing approximately 80,000 single nucleotide variants are observed per individual, of which approximately 250 encode substitutions not annotated in public databases. To further reduce this number, a powerful approach has been to compare affected cousin-pairs, in contrast to affected sibling-pairs, to reduce the number of alleles shared by descent (from approximately 125 to approximately 31 variants). In ‘pair-wise sharing analysis’ non-synonymous missense mutations that have a frequency less than the incidence of PD (<0.003), which are evolutionarily conserved and predicted as damaging, are prioritized as good candidates for follow-up. However, caution is warranted in comparative analyses: the phenocopy rate of late-onset PD is approximately 18% in families in which a monogenic cause of disease is already defined [77]. The strategy may also exclude causal or risk variants. For example, *SNCA* p.A53T was the first mutation identified in PD but is neither evolutionarily conserved nor predicted to be deleterious [56].

## Molecular pathways in PD

**Figure 1:**
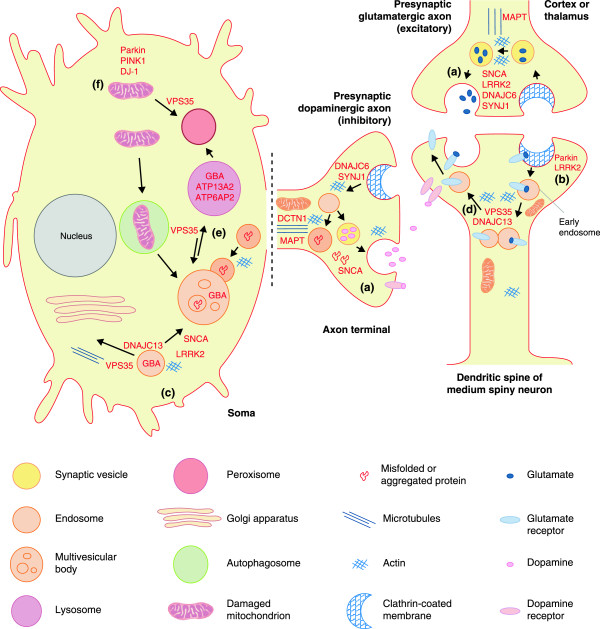
**Cellular processes implicated in familial late-onset, Lewy body parkinsonism.** In late onset parkinsonism, which is most reminiscent of Lewy body PD, a novel synthesis is emerging whereby regulatory steps in synaptic neurotransmission, receptor recycling, endosomal trafficking and lysosomal degradation are controlled by relatively few proteins. Studies in neurons and brain are limited but many reciprocal connections are apparent [8]. For example, **(a)** SNCA functions with heat shock chaperone Hsc70 and SNAP25 to promote membrane SNARE complex assembly and exocytosis in neurotransmission. **(b)** In mammalian cells, LRRK2 may also regulate cleavage of invaginated endocytic membrane through interaction with dynamin. Several other genes, including *DNAJC6* (with homology to *GAK*) and *SYNJ1*, encode proteins important in clathrin uncoating. In *Drosophila*, LRK1 (the homolog of mammalian LRRK1 and LRRK2) phosphorylates endophilin A to directly regulate endocytosis. In the mammalian striatum the expression levels of SNCA and endophilin A are reciprocally related. **(c)** In the endosome, VPS35 and the retromer cargo-selective-complex (CSC; comprising VPS35-VPS26 and VPS29) play an important role in membrane protein cargo sorting. The CSC is best described in endosome to trans-Golgi network retrieval in the soma, most specifically in recycling cation-independent mannose-6-phosphate receptor that traffics acidic hydrolases (including GBA) to the lysosome. However, recent studies in neurons have revealed that VPS35 and WASH are also crucial in protein recycling **(d)** of specific synaptic receptors from early endosome to plasma membrane, and **(e)** in the V-ATPase required for lysosomal acidification. Importantly, CSC trafficking can be mediated by sorting nexin interaction with dynactin p150^Glued^ (DCTN1), dynein, tau (MAPT) and microtubules, or via the WASH complex, DNAJC13 and actin polymerization. DNAJC13 was first described in endocytosis in *C. elegans*, rather than in post-endocytic trafficking, and like SNCA requires Hsc70 to function. LRRK2 has also been shown to interact with VPS35 and microtubules, and like SNCA may be intrinsically targeted to lysosomal membranes by protein motifs for chaperone-mediated autophagy. Clearance of insoluble SNCA aggregates is also mediated by the endosomal system and lysosomal degradation [153]. Several other lysosomal proteins, including ATP13A2 and ATP6AP2, have been implicated in atypical parkinsonism. EIF4G1, through mTOR regulation of protein translation, serves to balance autophagic activity and metabolism or ATP levels, whereas **(f)** PINK1 and Parkin are intrinsically involved in mitochondrial quality control in early onset parknsonism.

Many genetic components now appear to contribute to the pathogenesis of parkinsonism. It is largely unknown whether the proteins involved function in overlapping biological pathways, whose dysfunction results in the progressive loss of striatal dopaminergic innervation and death of midbrain nigral neurons. Nevertheless, some relationships, involving the perturbation of relatively few cellular systems, are apparent (Figure 1). The affected systems include synaptic transmission, endosomal trafficking, lysosomal-autophagy and energy metabolism or mitophagy.

### Synaptic transmission

Alpha-synuclein protein is found abundantly at the presynaptic terminals of neurons and is involved in synaptic release [13] (Figure 1). Monomeric forms of alpha-synuclein may contribute to endophilin-A1-related membrane curvature, facilitating both synaptic vesicle exo- and endocytosis [78]. A tetrameric conformation has been proposed for alpha-synuclein and this might provide a parsimonious explanation of how amino-terminal point mutations lead to the same functional deficits [79,80]. The conformation of alpha-synuclein has previously revealed physiologic interactions with mitochondria [81] and with presynaptic tubulovesicular or endosomal structures when alpha-synuclein is overexpressed in transgenic mice [82].

At the synapse, LRRK2 levels regulate glutamate transmission, dopamine-dependent plasticity and striatal signal transduction [83,84]. LRRK2 protein levels and mutant-specific phenotypes have long been observed in neuritic outgrowth (affecting branching and length) in primary cultures [85,86] and in neurogenesis *in vivo*[87]. In *Drosophila*, LRRK2 kinase has been shown to regulate the EndoA phosphorylation cycle, and pathogenic mutations appear to impede synaptic endocytosis [88] (Figure 1). LRRK2 protein is also reported to interact with the dynamin superfamily of GTPases, which mediate both membrane scission in clathrin-induced endocytosis and mitochondrial fission and fusion [89]. In *Caenorhabditis elegans*, knockout of LRK-1 (the single homolog of mammalian *LRRK1* and *LRRK2*) leads to impairment in presynaptic protein sorting and axonal trafficking [90]. Several important functions have also been ascribed to the LRRK2 protein complex in non-neuronal cells, including kidney cells [26,91], and in innate immunity [92].

Recessively inherited mutations in *DNAJC6* have recently been identified in juvenile parkinsonism [70]. *DNAJC6* encodes auxilin, a homolog of cyclin-G associated kinase (GAK; Table 1), which is preferentially expressed in neurons and involved in clathrin uncoating and synaptic vesicle recycling. Similarly, recessively inherited mutations in *SYNJ1*, encoding synaptojamin, that complexes with Hsc70 and auxilin, have been implicated in disease [72].

### Endosomal trafficking

Endosomal trafficking is a highly complex and dynamic cellular process whereby vesicles or cargos that are internalized at the plasma membrane are subsequently recycled, directly or via the trans-Golgi network, and targeted for degradation by lysosomal autophagy. Mutations in VPS35 and RME-8 (receptor-mediated endocytosis 8, also known as DNAJC13) were recently linked to late-onset Lewy body parkinsonism and directly implicate endosomal trafficking in disease pathogenesis [63,93]. Neurons have a critical need to recycle membrane receptors. This can be accomplished through the clathrin-independent retromer system, a tubulovesicular tripartite complex of VPS26 (vacuolar sorting protein 26), VPS29 and VPS35 that relies on sorting nexins to stipulate the destinations of specific cargos, such as neurotransmitter receptors. Multiple VPS35 subunits coalesce about FAM21, a subunit of the WASH (Wiskott-Aldrich syndrome protein and scar homolog) complex, to mediate dynamic actin remodeling [94]. RME-8 also binds sorting nexins and FAM21 to influence WASH and cargo trafficking [95]. VPS35 may also physically interact with LRRK2 and Rab7L1 (within the PM20D1 locus; Table 1) to influence these processes [96].

### Lysosomal autophagy

Lysosomes have an essential function in maintaining protein and organelle integrity within cells and impaired lysosomal function may play an important role in the pathogenesis of PD. Aggregated alpha-synuclein, in the form of Lewy neuritic or Lewy body inclusions, that fails to be degraded by proteosomal or lysosomal systems is the pathologic hallmark of PD. It is presently unknown whether the intracellular protein aggregation observed in most late-onset neurodegenerative diseases is a cause or consequence of dysfunction in these pathways [97]. The formation of intracellular aggregated alpha-synuclein or tau inclusions, albeit not a primary pathology, is also found in several ceroid lipofuscinosis disorders. These include glycolipid storage diseases such as Gaucher disease and Niemann-Pick type C that are most prevalent in Ashkenazi Jewish communities. Although the *GBA* (glucocerebrosidase gene) mutations are recessively inherited in Gaucher disease, heterozygote carriers have an increased prevalence of PD and dementia with Lewy bodies (Table 1) [98]. A lysosomal pathway for parkinsonism centered around ceramide metabolism has been hypothesized [99]. Loss of GBA activity increases intracellular glucosylceramide accumulation, resulting in decreased lysosomal degradation and subsequent accumulation of alpha-synuclein [100]. Whether the latter reflects impaired GBA trafficking from the endoplasmic reticulum and Golgi to lysosomes or whether it results directly from lysosomal dysfunction is unclear. In genetically engineered mice, *GBA* mutations promote alpha-synuclein accumulation in a dose- and time-dependent manner, with the animals developing Lewy-like pathology in the brain and associated motor and cognitive phenotypes. By contrast, loss of GBA activity results in neuronal ubiquitinopathy and formation of axonal spheroids, a phenotype that is shared with other lysosomal storage disorders prior to increased alpha-synuclein concentrations [101].

Two juvenile or early-onset forms of atypical parkinsonism result directly from mutations in lysosomal proteins. X-linked parkinsonism, with onset in males between 14 and 50 years of age and associated with post-mortem tau pathology, is a consequence of splicing or protein isoform deficits in *ATP6AP2* (encoding ATPase, H^+^ transporting, lysosomal accessory protein 2) [71]. Recessively inherited p*athogenic mutations in* ATP13A2 (ATPase type 13A2 gene) also result in impaired lysosomal proteolysis, leading to Kufor-Rakeb syndrome [102]. Patients and knock-out mice develop ceroid lipofuscin neuronal pathology, and the mice show concomitant upregulation of alpha-synuclein protein in the hippocampus [103]. Many genes are implicated in neurodegeneration with brain iron accumulation, including *ATP13A2*, *PLA2G6*, *PANK2*, *C19orf12*, *FA2H*, *WDR45*, *FTL*, *CP*, and *DCAF17*[104].

### Mitochondrial metabolism

The earliest link between mitochondrial dysfunction and parkinsonism was observed in illicit drug users. MPTP (1-methyi-4-phenyl-1,2,3,6-tetrahydropyridine) is specifically transported into dopamine neurons via the dopamine transporter and is then oxidized into toxic MPP^+^, a non-competitive complex inhibitor of the electron transport chain [105]. Deficits of mitochondrial complex I have been noted in idiopathic PD [106], although evidence from direct sequencing studies of normal brain has proven equivocal. Mitochondrial mutations in humans lead to several neuromuscular disorders [107]. While the majority are not associated with parkinsonism, similar movement disorders with or without chronic progressive ophthalmoplegia can be caused by mutations in mitochondrial DNA polymerase γ (POLG), a proofreading enzyme. Mouse models with defective POLG exhibit premature ageing whereas older homozygous, but not heterozygous, POLG mice show significant reductions in striatal dopaminergic terminals as well as deficits in motor function [108].

The importance of mitochondria in parkinsonism is highlighted by the identification of mutations in several genes within a common pathway for mitophagy. Mutations in the *PARK2* (*parkin*) gene result in a recessive form of early-onset parkinsonism [109]. Parkin protein was first described as a proteosomal E3 ubiquitin ligase responsible for K48 substrate polyubiquination (targeting to the proteosome) and K63 monoubiquination (for signaling) [110]. In addition, parkin may have several physiological roles in neurons - for example, in Eps15 monoubiquination [111] and in the regulation of neuronal apoptosis as part of a SCF-like complex [112]. Most highlighted is the role of parkin in regulating the degradation of depolarized or uncoupled mitochondria, in concert with PINK1 (Pten-induced kinase 1) and FBXO7 (F-box domain-containing protein), which are also genes implicated in recessive early-onset parkinsonism [68,113]. *Drosophila* parkin and *PINK1* knockout models exhibit similar mitochondrial and wing phenotypes, and a series of elegant experiments has demonstrated that PINK1 is required for the recruitment of parkin to mitochondria [114,115]. The crystal structure of parkin has now been solved [116] and PINK1 has been shown to phosphorylate ubiquitin required for parkin’s activation [117]. Two recent RNA interference screens have identified upstream regulators of mitophagy, albeit with limited overlap [118,119]. These include TOMM7, essential for stabilizing PINK1 on the outer mitochondrial membrane; HSPA1L and BAG4, which may help to regulate parkin translocation to mitochondria; and SIAH3, which is localized to mitochondria and inhibits PINK1 after mitochondrial damage, thereby reducing parkin translocation. Hexokinase activity, occurring downstream of Akt but upstream of PINK1, may also be required in the recruitment of parkin to depolarized mitochondria [119]. STOML2, mitofusin1/2, GRP75, HSP60, LRPPRC, and TUFM have been nominated as downstream targets of the PINK1/parkin pathway [120-122]. DJ-1 mutations, which result in early-onset parkinsonism [123], may also regulate PINK1-dependent parkin translocation to depolarized mitochondria [124]. DJ-1 deficiency leads to altered mitochondrial morphology and increased levels of reactive oxygen species (ROS) [124]. Traditionally, knock-out mouse models of parkin, PINK1 or DJ-1 result in mitochondrial dysfunction [125] but do not develop the locomotor phenotype of parkinsonism, nigral neuronal loss or Lewy-body pathology; rather they have elevated dopaminergic tone due to deficits in D2 presynaptic regulation of release [126,127]. However, a recent conditional parkin knockout mouse model demonstrated progressive loss of dopamine neurons in a PARIS-dependent pathway [128]. Thus, protein components of the parkin/PINK1 mitochondrial pathways remain plausible therapeutic targets for human carriers of these mutations and potentially for idiopathic PD.

While mitochondrial mutations and proteins involved in mitophagy have been directly implicated in parkinsonism, there is accumulating albeit indirect evidence that mitochondrial function is central to disease pathogenesis and/or progression. For example, alpha-synuclein overexpression may impair mitochondrial activity, thereby accumulating mitochondrial DNA damage and degeneration, ultimately resulting in neuronal death [129]. For LRRK2, mitochondrial pathology was observed in human dopaminergic neurons derived from inducible pluripotent cell lines of *LRRK2* p.G2019S carriers [130] and in aging p.G2019S transgenic mice [131]. In primary cortical neurons, overexpression of wild-type LRRK2 and of pathogenic mutant LRRK2 proteins both increased recruitment of mitochondrial dynamin-like protein to fragmenting mitochondria [132].

## Conclusions and future directions

Idiopathic PD, albeit a sporadic disorder with low heritability, now appears to have a significant genetic component. Many genes have been identified and several more will probably emerge from a variety of complementary approaches. Most immediately, targeted genomic sequencing might identify functional variants in GWAS-associated loci, whereas additional GWAS in non-Caucasian populations might identify novel loci. WES in families with parkinsonism is also a pragmatic step to identify more genes using a concordant ‘pairwise’ approach. Ultimately, collaborative pooled analysis of exome data might facilitate association analysis; power estimates suggest that as few as 50 exomes may be sufficient to identify a novel locus for recessive disease, although 10,000 are likely to be required to identify a novel dominant gene mutation [133]. Nevertheless, segregation of rare mutations with disease and/or functional studies will be needed. Inevitably, with less penetrant variants, the results from these approaches will become increasingly difficult to interpret. Hence, rather than focusing on PD as the trait, more investment may be warranted on longitudinal studies. In addition, better characterization of trait components, such as cognition in PD, symptom progression and response to medication, would enable further genetic variability to be identified. Similar analyses in more genetically homogeneous populations (employing linkage, association and genome sequencing) and in sample series or pedigrees of sufficient size and structure may enable the joint contribution of genes and environment to be assessed meaningfully. We suggest that a genetic predisposition to PD should not be considered ‘causal’, rather disease reflects chronic molecular dysfunction and the failure of age-associated compensation.

To date, four biological pathways have been implicated in familial parkinsonism: synaptic neurotransmission, endosomal trafficking, lysosomal autophagy and mitochondrial metabolism. Direct interactions between genetic components and these pathways are emerging, whether *VPS35* and *RME-8* in late onset PD, or *parkin* and *PINK1* in mitophagy in early onset parkinsonism. Although the processes highlighted may be viewed separately, several employ the same protein machinery and may be temporally and functionally related. For example, synaptic dysfunction, resulting from or leading to alpha-synucleinopathy, impairs the balance of exo- and endocytosis, neurotransmission and early endosomal receptor recycling. These changes will alter flux through the endosomal pathway and ultimately place demand on autophagy and lysosomal fusion with multivesicular bodies. Many of the same proteins are involved in more than one of the four pathways; for example, VPS35 directly affects both early-endosome receptor recycling in dendritic spines and lysosomal ATPase recycling from multivesicular bodies [134]. Similarly, LRRK2 appears to be centrally involved in neurite outgrowth and in membrane protein cargo sorting and trafficking, interacts with dynamin GTPases, and may regulate endophilin phosphorylation, membrane scission and endocytosis. VPS35, RME-8 and potentially LRRK2 coordinate the WASH complex in specific actin networks underlying membrane deformation, tubulation and cargo trafficking. The caveat is that many of the biological insights may be model specific, or have yet to be performed in vertebrate systems in post-mitotic neurons or in brain.

In conclusion, genomic and genetic investigations should continue to be a main priority for future research. Knowledge of the pathogenic pathways underlying the etiology and ontology of PD clearly facilitates an understanding of common protein components and central processes that are crucial for therapeutic development. Our understanding of the normal physiology of the brain, of specific neuronal populations, protein pathways and the function of individual proteins, is rudimentary. When it comes to formulating a molecular synthesis of the pathways involved in parkinsonism, genetic insights may be unbiased and unequivocal but those insights must be carefully weighted. Genetic association is far from causation and much work is required to understand the specific contribution of GWAS, let alone to translate the information from such studies into novel treatments to slow or halt the progression of idiopathic PD. In interpreting linkage and exome studies, the phenotypes of patients and families must be carefully considered. The heterogeneity of parkinsonism is considerable and there are many forms that may not be etiologically related. Arguably, findings from familial late-onset Lewy-body parkinsonism may be more relevant to idiopathic PD than those from atypical and/or early-onset forms. Brain pathology, long required for a definite diagnosis, appears increasingly pleomorphic in genetically defined disease, even for the same mutation in the same family. Such alternative pathologic outcomes become more intriguing as specific molecular deficits in membrane protein sorting and cargo trafficking are revealed. That aggregate alpha-synuclein and tau pathologies may be seeded and transmissible throughout the cerebrum presents an attractive means to explore Braak staging (a regional and temporal scheme for the progression of these inclusion body pathologies) and vulnerable cell populations in specific genetic backgrounds. Much neuroscience in PD was derived from model systems based on toxin administration, and may not accurately reflect the human condition. Why the *substantia nigra pars compacta* is selectively lost in PD remains enigmatic, but through human genetics we now have relevant molecular targets and tools to investigate this. With such advances, therapeutic prospects for disease modification (neuroprotection) should be viewed with more optimism [135].

## Box 1. Parkinsonism and Parkinson’s disease

Parkinsonism is used as an adjective to describe a movement disorder generally consisting of one of more features of tremor, bradykinesia and rigidity. The most common forms are drug-induced (for example, as a side-effect of antipsychotic neuroleptics), or vascular resulting from a stroke. When used as an adjective, the term parkinsonism does not imply that the symptoms are progressive or neurodegenerative, or that they are associated with a specific neuropathology. Parkinsonism may not respond to levodopa therapy. PD itself has the same core triad of symptoms, but it begins asymmetrically, one side of the body being more profoundly affected than the other, and postural instability is typically a feature. In PD, symptoms respond well to levodopa replacement therapy, and typically worsen with disease duration despite medication. Symptoms become bilateral in advanced PD, and dopaminergic imaging, using modalities such as DaTscan, highlights the same pattern of progression.

PD, but not parkinsonism, is often associated with a variety of autonomic problems (constipation, bowel and bladder incontinence, drooling), cognitive dysfunctions (from mild impairments in executive function to dementia), motor problems (from dystonia to dyskinesia), neuropsychiatric symptoms (mood disorders, such as depression to impulse control), sensory problems (from unexplained pain syndromes to hyposmia or anosmia) and sleep disorders (daytime sleepiness to REM sleep behavior disorder). Several of these problems, including dyskinesia, mood disorders and daytime sleepiness, may be associated with levodopa therapy or the use of dopamine agonists.

A definite diagnosis of PD is reserved for patients with Lewy body pathology and neuronal loss in the midbrain, which is often more widely distributed to involve the myenteric plexus, vagus and olfactory bulb. By contrast, parkinsonism may be associated with a variety of pathologies, including neurofibrillary tangles (such as in progressive supranuclear palsy, corticobasal ganglionic degeneration, or parkinsonism-dementia complex of Guam), to the predominant oligodendroglial alpha-synuclein pathology of multiple system atrophy, or predominant cortical Lewy pathology associated with dementia with Lewy bodies.

## Box 2. Genetic insights and evolving neuroscience

**1997** A missense substitution, p.A53T, is discovered in the gene encoding alpha-synuclein (*SNCA*) in a family from Contursi, Italy whose members were susceptible to autosomal dominant, late-onset parkinsonism [56]. Alpha-synuclein, then known as ‘Non-amyloid component of plaques’ (NACP), provides a link between PD and Alzheimer’s disease. Whether alpha-synuclein monomers, oligomers or fibrils are the toxic species becomes a topic for debate. Physiologically, the Zebra Finch homolog of *SNCA* is found to be required for song learning, pre-synaptic plasticity, and vesicle trafficking in neurotransmission.

Alpha-synuclein immunohistochemistry reveals much more occult Lewy pathology than had been visualized previously by hematoxylin and eosin (H&E) staining, and replaced the use of PGP9.5 (an antibody for Ubiquitin C-terminal hydrolase1 (UCHL1)) [136]. It is argued that familial and idiopathic forms of parkinsonism are the same disease, with similar ontology. Nevertheless, epidemiologists claim that the etiology of late-onset PD is environmental, rather than due to a genetic predisposition, supported by the results of twin studies. Further debate centers on whether ubiqinated Lewy body ‘aggresomes’ are pathologic or protective.

**1998** Homozygous parkin deletions are linked to recessively inherited juvenile and early-onset parkinsonism, albeit without documented Lewy pathology [109]. Parkin mutations soon explain around 15% of all early-onset parkinsonism (in those younger than 45 years) [137]. As the parkin mutations affect a ubiquitin E3 ligase, the field focuses on proteasome inhibition, attempting to identify parkin’s substrates and to nominate the toxic species. Patients with parkin mutations and Lewy or tau pathology have subsequently been described [138,139]. The crystal structure of the ubiquitin ligase reveals how its activity is regulated [116].

**1999-2001** Candidate gene studies of polymorphic variability in *SNCA* and microtubule-associated protein tau (*MAPT*) highlight the roles of these genes in idiopathic, late-onset PD [57,140,141]. *MAPT* is implicated in progressive supranuclear palsy, in part because of the discovery of splicing mutations in another 4R-tauopathy, frontotemporal dementia with parkinsonism linked to chromosome 17 (FTDP-17) [44]. Large-scale GWAS provide further confirmation [9,52,55,142].

**2003** *DJ-1* mutations that were discovered in early-onset parkinsonism [123] highlight a role for oxidative stress in PD. The findings help to justify the use of toxin-based models using 1-methyl-4-phenyl-1,2,3,6-tetrahydropyridine (MPTP) as an analog of the herbicide paraquat or the pesticide rotenone.

**2003-2004** *SNCA* triplication and duplication mutations demonstrate a dose-dependent relationship between expression and pathogenicity [58,143-146] that is now supported by several mouse models. Lowering *SNCA* RNA and/or SNCA protein expression is nominated as a therapeutic target, supported by *in vivo* studies.

**2004** Recessively inherited mutations are identified in the Pten-induced kinase 1 gene (*PINK1*) in early-onset parkinsonism [147]. By 2006, PINK1 was found to regulate Parkin recruitment and mitophagy [114,115]. *FBXO7* is within the same pathway, pointing to mitochondrial maintenance as a therapeutic opportunity [68]. Hexokinase is identified through interaction screens as an upstream component of the pathway [119]. PINK1 is shown to phosphorylate ubiquitin to activate parkin [117].

**2004-2005** Dominantly inherited mutations are identified in the *LRRK2* gene in late-onset PD [32]. In 2005, *LRRK2* p.G2019S was linked to PD in Norwegian families [148], and found to be the major determinant of sporadic PD in Ashkenazi Jews and North-African Berbers. Kinase inhibition is nominated as a therapeutic strategy. Pleomorphic alpha-synuclein, 4R-tau or ubiquitin pathologies in affected carriers suggest that Lewy pathology should not be required for a definite diagnosis of PD [32]. LRRK2 is implicated in protein sorting or trafficking and in autophagy. Polymorphic variants in *LRRK2* are found to lower or increase the risk of sporadic PD [34].

**2006** *ATP13A2* recessive mutations are identified in juvenile and early-onset parkinsonism [102]. Several lysosome-associated proteins have subsequently been linked to rare and rather atypical forms, including *ATP1A3* in rapid-onset parkinsonism-dystonia, and most recently *ATP6AP2*[71]. Polymorphic variants in the glucocerebrosidase gene *GBA1* are reproducibly associated with late-onset PD, highlighting a role for endosomal trafficking and lysosomal function [98].

**2009** GWAS studies in PD confirm associations with *SNCA* and *MAPT*, and find evidence for additional loci [50-52].

**2011** *VPS35* D620N is linked to dominant late-onset PD [63]. Many families have the same, albeit *de novo*, substitution that supports pathogenicity. By contrast, mutations in *EIF4G1* R1205H require further genetic and functional support for their assignment [149].

**2013-2014** Mutations in *DNAJC13*, along with *VPS35*, in late-onset Lewy-body PD further highlight involvement of the retromer-WASH complex, endosomal protein sorting and trafficking [63,64,93].

**2014** Further meta-analysis of GWAS supports known and novel loci. Nevertheless, for most loci the precise gene and underlying variability remain elusive.

## Abbreviations

ATP13A2: ATPase type 13A2 gene; ATP6AP2: ATPase, H^+^ transporting, lysosomal accessory protein 2; CSC: cargo-selective-complex; DBS: deep brain stimulation; EIF4G1: elongation initiation factor 4 gamma 1; FBXO7: F-box domain-containing protein 7; FTDP-17: frontotemporal dementia with parkinsonism linked to chromosome 17; GAK: cyclin-G associated kinase; *GBA*: glucocerebrosidease gene; GWAS: genome-wide association study; H&E staining: hematoxylin and eosin staining; LRRK2: leucine-rich repeat kinase 2 protein; MAPT: microtubule-associated protein tau; MPTP: 1-methyi-4-phenyl-1,2,3,6-tetrahydropyridine; NACP: non-amyloid component of plaques; NGS: next-generation sequencing; OMIM: Online Mendelian Inheritance in Man; PD: Parkinson’s disease; POLG: DNA polymerase γ; RME-8: receptor-mediated endocytosis 8; ROS: reactive oxygen species; SN: *substantial nigra pars compacta*; *SNCA*: alpha-synuclein gene; SNP: single nucleotide polymorphism; UCHL1: Ubiquitin C-terminal hydrolase1; UTR: untranslated region; VPS35: vacuolar sorting protein 35; WASH: Wiskott-Aldrich syndrome protein and scar homolog; WES: whole-exome sequencing; WGS: whole-genome sequencing.

## Competing interests

The authors declare that they have no competing interests.
